# Chemical Characterization and Antihyperlipidemic Potential of *Canarium schweinfurthii* Engl. Fruit Pulp Oil in *Wistar* Rats

**DOI:** 10.1002/fsn3.71474

**Published:** 2026-02-12

**Authors:** Archelle Arnellie Abaoabo Foudjin, Hermine Tsafack Doungue, Stephano Tambo Tene, Ronice Zokou, Tekou Florian Amel, Geradin Joel Tagne Tueguem, Anne Pascale Kengne Nouemsi

**Affiliations:** ^1^ Research Unit of Biochemistry of Medicinal Plants, Food Science and Nutrition, Department of Biochemistry, Faculty of Science University of Dschang Dschang Cameroon; ^2^ Research Unit of Analytical Chemistry Laboratory, Gembloux Agro‐Bio Tech of the University of Liege Gembloux Belgium; ^3^ Laboratory of Nutrition and Nutritional Biochemistry, Department of Biochemistry, Faculty of Science University of Yaounde 1 Yaoundé Cameroon

**Keywords:** antihyperlipidemic activity, antioxidant activity, *Canarium schweinfurthii*, chemical characterization, dyslipidemia, pulp oil

## Abstract

The consumption of oleaginous fruits, with proven composition of unsaturated fatty acids and biological properties, can prevent dyslipidemia. To assess the antihyperlipidemic potential of *Canarium schweinfurthii* Engl. pulp oil. *Canarium schweinfurthii* pulp oil was obtained by hexane maceration, and characterized by the determination of fatty acid profile, chemical quality and in vitro antioxidant activity. The antioxidant and antihyperlipidemic capacities of the oil on 36 rats were evaluated. A hypercaloric diet over 42 days facilitated the induction of dyslipidemia. Simultaneously, 1 and 2 mL/kg body weight of oil were administered by gavage. After sacrifice, serum was collected, then lipid profile and antioxidant parameters were assessed. The oil from the pulp of *Canarium schweinfurthii* contains mainly palmitic (39.68%), oleic (28.13%) and linoleic acids (28.19%). Iodine, peroxide, thiobarbituric acid, anisidine and total oxidation values were, 54.06 ± 0.70 g I2/100 g, 4.02 ± 0.21 meq O2/kg, 0.44 ± 0.02 meq MDA/kg, 1.33 ± 0.00 and 9.37 ± 0.43 respectively. The value of EC50 was 11.81 μg/mL. Compared with the diseased group, 2 mL/kg dose of oil significantly reduced (*p* < 0.05) TAG (93.83 ± 1.69; 75.44 ± 4.57), total cholesterol (72.89 ± 3.59; 62.69 ± 6.24), LDL (15.52 ± 5.72; 20.27 ± 4.92), VLDL (18.76 ± 0.91; 15.08 ± 1.08), total lipids (268.24 ± 7.70; 226.13 ± 18.95) in males and females, respectively (mg/dL). This dose also reduced the atherosclerosis index (1.88 ± 0.17; 2.29 ± 0.27). A significant increase (*p* < 0.05) of HDL levels (38.61 ± 1.63 mg/dL; 27.33 ± 3.28 mg/dL), enzymatic activity of SOD (85.00% ± 17.32%; 91.66% ± 2.88%) and catalase (10.67 ± 0.36 μM/min/mL; 10.85 ± 0.47 μM/min/mL) were observed for both sexes. *Canarium schweinfurthii* oil is of good quality with high antiradical activity, which improves antioxidant defense and antihyperlipidemic activity in rats.

## Graphical abstract

The consumption of fruits like oleaginous fruits with their composition of unsaturated fatty acids whose biological properties have been proven, can prevent dyslipidaemia was to assess the antihyperlipidaemic potential of *Canarium schweinfurthii Engl*. pulp oil. *Canarium schweinfurthii* pulp oil was characterized, the antioxidant and antihyperlipidaemic capacities of the oil were evaluated on 36 rats. *Canarium schweinfurthii* oil is of good quality with high antiradical activity, which improves antioxidant defence and antihyperlipidaemic activity in rats.

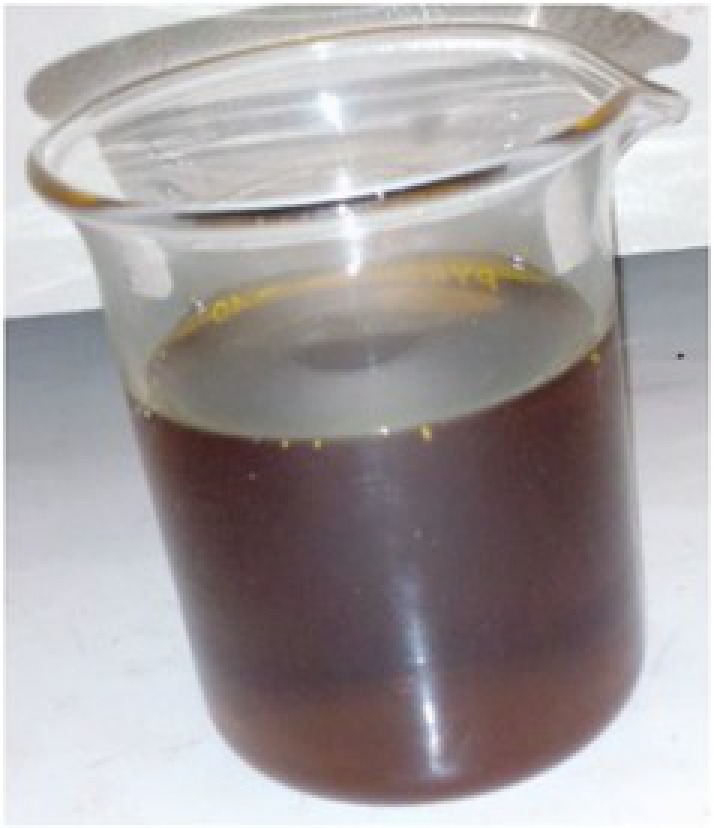

## 1 Introduction

Dyslipidemias are one of the so‐called modifiable cardiovascular risk factors, and their control provides a strategy for managing cardiovascular diseases (Hauguel‐Moreau 2023). This disease is defined as a metabolic disorder of lipoproteins, resulting in an abnormal decrease or increase of circulating lipid fractions such as triacylglycerols (TAGs), total cholesterol (TC), very low‐density lipoproteins (VLDL), low‐density lipoprotein (LDL), and high‐density lipoprotein (HDL) in a fasting lipid panel (Souza et al. 2017; Ali et al. 2019). Their prevalence rose from 43% to 70.2% between 1996 and 2014 in Cameroon (Thornalley et al. 1996; Bekolo et al. 2014). Their causes can include industrialization and globalization, which led to a rapid evolution of poor eating habits and lifestyle changes (World Health Organization 2014; Kamdem et al. 2018). Indeed, processed foods are sources of saturated fatty acids (SFAs), trans polyunsaturated fatty acids, and oxidation products, which are risk factors for dyslipidemias (World Health Organization 2002; Mbundu et al. 2018).

Dyslipidemia is generally managed by medication using statins, niacins, and fibrates designed to lower blood concentrations of total cholesterol, LDL, and/or triacylglycerols, and increase those of HDL (Handayani et al. 2021). However, the high cost of these treatments, side effects that they cause (myalgia, muscle weakness, renal failure, gastric ulcers, hyperuricemia), and the fact that they are prescribed only to patients whose cardiovascular risk associated with dyslipidemia is either intermediate or high are all factors limiting this drug‐based management. This is why particular emphasis is increasingly being placed on dietary therapy in view of the availability of foods, their relatively low costs, and a reduction of harmful side effects in addition to the presence of compounds with proven bioactive properties. Several studies have shown that eating fruits and vegetables has a beneficial effect on reducing the risk of dyslipidemia, due to their phenolic compound content and omega 3 and omega 6 fatty acids (Takam et al. 2019; Kengne et al. 2020).

Studies carried out on *Canarium schweinfurthii Engl*, a tree in the Burseraceae family, have shown that it produces fruits commonly known as “black fruits.” Their production is still traditional, very limited and used for human nutrition and health (Tsewoue et al. 2019). Ayoade et al. (2017) showed that the fruits contained 17.24% of carbohydrates, 12.67% of proteins and 34.83% of lipids. Its oil contains mainly palmitic (50.51%), oleic (32.02%), and linoleic (15.18%) acids. Its composition in UFA and antioxidants gives it biological properties (Anyalogbu Ernest et al. 2017). This fruit has been studied for its properties in human health such as anticancer, antioxidant, antimicrobial, and antidiabetic (Tcheghebe et al. 2016).

Due to its unsaturated fatty acid composition, *C. schweinfurthii* pulp oil is thought to have antioxidant and antihyperlipidemic properties, and thus to help prevent dyslipidemia. The aim of this study is to assess the antihyperlipidemic potential of *Canarium schweinfurthii* Engl. pulp oil.

## 2 Material and Methods

### 2.1 Material

#### 2.1.1 Plant Material

**FIGURE 1:**
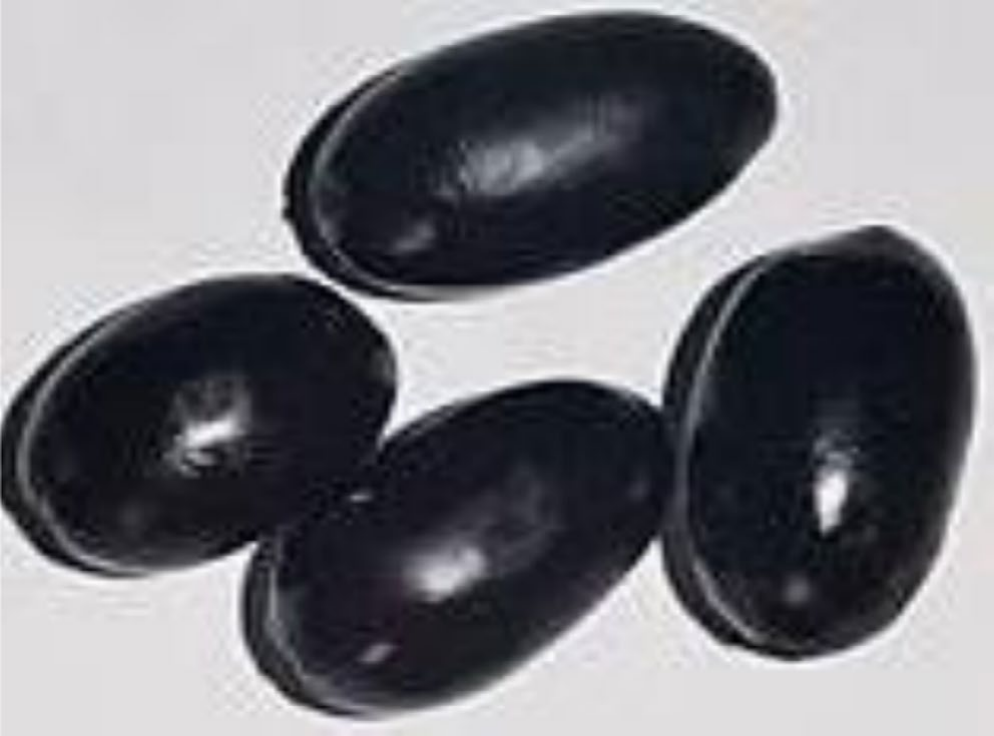
*Canarium schweinfurthii* ripe berries.

The fruits of *C. schweinfurthii* , commonly known as “black fruits,” from Bangang in Bamboutos department, West Cameroon were collected in March 2021 (Figure 1).

#### 2.1.2 Animal Material

Albino rats of *Wistar* strain were bred in the Biochemistry Department's animal house at the University of Dschang. They were given water ad libitum in addition to the feed prepared according to the protocol described by Telefo (1998) under a 12 h light/dark cycle. They were treated in accordance with OECD principles, which prioritize animal welfare throughout the study. The number of rats used in the experiments was limited to the minimum necessary, and methods were employed to minimize suffering and pain (OECD 2023). An ethical clearance for animal handling and care was obtained from the university of Buea‐institutional animal care and use committee permit number: UB‐IACUC No 01/2024.

## 3 Methods

The fruits (10 kg) that have been ripening for about 10 months, the time required for complete ripening after fruiting, were used. They were sorted, washed with clean water, and the pits containing the seeds removed with a stainless‐steel knife. The pulps were dried in a Heraeus ventilated air oven at 45°C for 48 h, and powder was obtained by grinding using a Delta domestic blender. The powder was stored in the desiccator for future analysis.

### 3.1 Characterization of *Canarium schweinfurthii* Pulp Oil

#### 3.1.1 Lipid Content

The Soxhlet method with hexane for 8 h described by Association of Official Analytical Chemists (AOAC) (1990) was used to determine the lipid content.

#### 3.1.2 Lipid Extraction

The hexane maceration method described by the protocol proposed by Womeni et al. (2010) was used to extract *C. schweinfurthii* pulp oil. The mixture, filtered through Whatman No. 1 paper after 48 h, was concentrated in a rotary evaporator at 50°C. A ventilated air oven at 50°C was used to dry the oil for 2 h to remove all hexane residues. The oil was stored in opaque glass bottles and kept in the freezer at 4°C for subsequent analysis.

#### 3.1.3 Fatty Acid Profile of *Canarium schweinfurthii* Pulp Oil

Transesterification of fatty acids was carried out using the method described by Jariyasopit et al. (2021). To assess the fatty acid profile of *M. arboreus* pulp oil, the fatty acid methyl esters (FAMEs) formed were analyzed by gas chromatography coupled to a flame ionization detector from Interscience Thermo Electron Corporation (Science Park Einstein/1348 Louvain La‐neuve, Belgium) equipped with an AI 3000 automatic injector (Thermo Electron Corporation) and a column Stabil Wax‐DA (30 m × 0.25 mm ID × 0.25 μm inner diameter film). The oven temperature was programmed to range from 50°C to 250°C at a rate of 3°C/min. Injector and detector temperatures were 250°C and 270°C, respectively. Split mode was 1 μL injection and a ratio of 10:1. The gas flow's parameters were 250°C, 300 mL/min of air, and 25 cm/s of helium. The peaks were identified based on their retention time by comparison with the SUPELCO standard consisting of 37 FAMEs.

#### 3.1.4 Antioxidant Activity of *Canarium schweinfurthii* Pulp Oil

The method described by Mensor et al. (2001) was used to evaluate the antioxidant activity of *C. schweinfurthii* oil by the ability to trap the 2,2‐diphenyl‐1‐picrylhydrazyl radical (DPPH‐). The effective concentration 50 (EC50) was also calculated.

#### 3.1.5 Quality Indices of *Canarium schweinfurthii* Pulp Oil

The quality of an oil can be influenced by many factors which can indicate its degree of deterioration. It is determined by the evaluation of quality indices like: acid and iodine values AFNOR (Association Française de Normalisation) (1981); peroxide value (International Dairy Federation Standards 1991); thiobarbituric acid value (Draper and Hadley 1990); anisidine value (AOCS 2003); and total oxidation value (Shahidi and Wanasundara 2008).

#### 3.1.6 Nutritional Quality Indices of *Canarium schweinfurthii* Pulp Oil

The following calculation formulas shown in Table 1 were used to determine the nutritional quality indices of *C. schweinfurthii* pulp oil according to the methods described by Chen and Liu (2020); Mgbechidinma et al. (2023); Rahman et al. (2023).

**TABLE 1:** Calculation formulas of nutritional quality indices.

Nutritional quality indices	Calculation formula
∑SFA	Total saturated fatty acids
∑UFA	Total unsaturated fatty acids
∑MUFA	Total monounsaturated fatty acids
∑PUFA	Total polyunsaturated fatty acids
PUFA/SFA	$\frac{\text{PUFA}}{\mathrm{SFA}\;\text{ratio}}$
∑(*n* − 6)	Total omega 6
∑(*n* − 3)	Total omega 3
Linoleic acid/α linolenic acid	$\frac{\text{Linoleic acid}}{\alpha \;\text{linolenic acid ratio}}$
Atherogenic Index (AI)	$\frac{C12:0+\left(4x\;C14:0\right)+C16:0)}{\sum \mathrm{UFA}}$
Thrombogenic Index (TI)	$\frac{\left(C14:0+C16:0+C18:0\right)}{(\left(0.5\text{xΣMUFA}\right)+\left(0.5x\sum \left(n-6\right)\right)+(3x\sum \left(n-6\right)+\left(\sum \left(n-3\right)/\sum \left(n-6\right)\right)}$
Hypocholesterolemic/hypercholesterolemic ratio	$\frac{(\mathrm{cis}-C18:1+\text{ΣPUFA}}{\left(C12:0+C14:0+C16:0\right)}$
Health‐promoting index	$\frac{\text{ΣUFA}}{\left(C12:0+\left(4x\;C14:0\right)+C16:0\right)}$
Nutritive value index	$\frac{\left(C18:0+C18:1\right)}{C16:1}$

### 3.2 Experimental Protocol and Induction of Dyslipidemia

Dyslipidemia was induced for 42 days by consumption of a hypercaloric diet. It consisted of basic food, pork fat rendered in previously bleached unrefined palm oil. The protocol of Sayin et al. (2016) was used with slight modifications. The rendered fat‐bleached palm oil mixture was prepared in the ratio 5:3 volume to mass. The composition of each experimental diet is presented in Table 2.

**TABLE 2:** Composition of experimental diets.

Ingredients	Standard diet (per 1000 g)	Hyperlipidaemic diet (per 1000 g)
Corn flour	680	510
Soy flour	200	150
Fish meal	100	75
Bone meal	10	7,5
Cooking salt	8	6
Refined palm oil	1.25	0
Rendered pork fat	0	250.75
Vitamin complex	0.75	0.75

A total of 36 *Wistar* albino rats aged 120 days were grouped into six groups (three males, three females) and were used in this study. Each rat was placed in a cage and subjected to a nycthemeral cycle throughout the experimental period. The rats were fed on a standard diet and water *ad libitum* for 1 week to stabilize their metabolism before the onset of dyslipidemia induction. After this acclimatization period, 5 groups (GG, GC1, GC2, GO1, and GO2) were fed a high‐calorie diet for 42 days, while the last group (GS) was fed on a basic diet. While maintaining a high‐fat diet, the rats received two doses of *C. schweinfurthii* pulp oil (GC1, GC2) and olive oil (GO1, GO2) orally via gavage tube; the latter was used as the reference oil for the prevention of dyslipidemia. These rats were fed 40 g of feed previously kneaded with water and the various oils at doses of 1 and 2 mL/kg body weight once a day for 42 days by gavage. On the other hand, the GS and GG groups received distilled water at a dose of 1 mL/kg body weight, again by gavage. The number of rats used in the experiments was limited to the minimum necessary, and methods were employed to minimize suffering and pain (OECD 2023).

Briefly, they were distributed as shown in Table 3.

**TABLE 3:** Distribution of rats.

Group	Diet
GS	Basic feed + 1 mL/kg body weight/day distilled water
GG	High‐calorie feed + 1 mL/kg body weight/day distilled water
GC1	High‐calorie feed + 1 mL/kg body weight/day of *C. schweinfurthii* pulp oil
GC2	High‐calorie feed + 2 mL/kg body weight/day of *C. schweinfurthii* pulp oil
GO1	High‐calorie food + 1 mL/kg of body weight/day of olive oil
GO2	High‐calorie food + 2 mL/kg of body weight/day of olive oil

Abbreviations: GC1 = Test rats received 1 mL/kg of body weight of *C. schweinfurthii* oil, GC2 = Test rats received 2 mL/kg of body weight of *C. schweinfurthii* oil, GG = Negative controls, GO1 = Positive controls received 1 mL/kg of body weight of olive oil, GO2 = Positive controls received 2 mL/kg of body weight of olive oilGS = Neutral controls.

### 3.3 In Vivo Antihyperlipidemic Potential of *Canarium schweinfurthii* Pulp Oil

#### 3.3.1 Sacrifice and Serum Preparation

After 42 days of test, the rats were subjected to a 12‐h food fast. They were then anesthetized by intramuscular injection of ketamine hydrochloride at a dose of 10 mg/kg body weight (Jalde et al. 2016). Blood samples were collected using syringes by cardiac puncture, placed in dry tubes and centrifuged at 3500 rpm for 15 min. The resulting sera were placed into Eppendorfs tubes and stored at −20°C for subsequent biochemical analysis.

#### 3.3.2 Determination of Serum Biochemical Parameters

The serum lipid profile of the rats was determined by enzymatic colorimetry using commercial Dutch Diagnostics kits. The method described by Trinder (1969) was used to determine HDL, total cholesterol, and triacylglycerol levels, with the ratio (TAG/5) representing the VLDL level. The LDL fraction and the atherosclerosis index (AI) were determined by formulae (1) and (2), respectively (Friedewald et al. 1972; Trinder 1969).

(1) $$\mathrm{LDL}=\text{Total Cholestérol}–\mathrm{HDL}–\mathrm{TAG}/5\;\left(g/L\right)$$

(2) $$\mathrm{AI}=\frac{\mathrm{TC}}{\mathrm{HDL}}$$

#### 3.3.3 Antioxidant Properties of *Canarium schweinfurthii* Pulp Oil

Superoxide Dismutase (SOD) and catalase activity were determined using the method described by Sun et al. (1988) and Sinha (1972), respectively.

### 3.4 Data Analysis

The results were presented in the form of mean ± standard deviations of 3 repetitions and calculated using Microsoft Office Excel 2013. Statistical analyses were performed using IBM SPSS software, version 22. Analysis of variance (ANOVA) at the 5% probability threshold with a Duncan's post hoc test was performed to compare the means of lipid profile parameters and markers of oxidative stress of experimental rats.

## 4 Results

### 4.1 Characterization of *Canarium schweinfurthii* Pulp Oil

**FIGURE 2:**
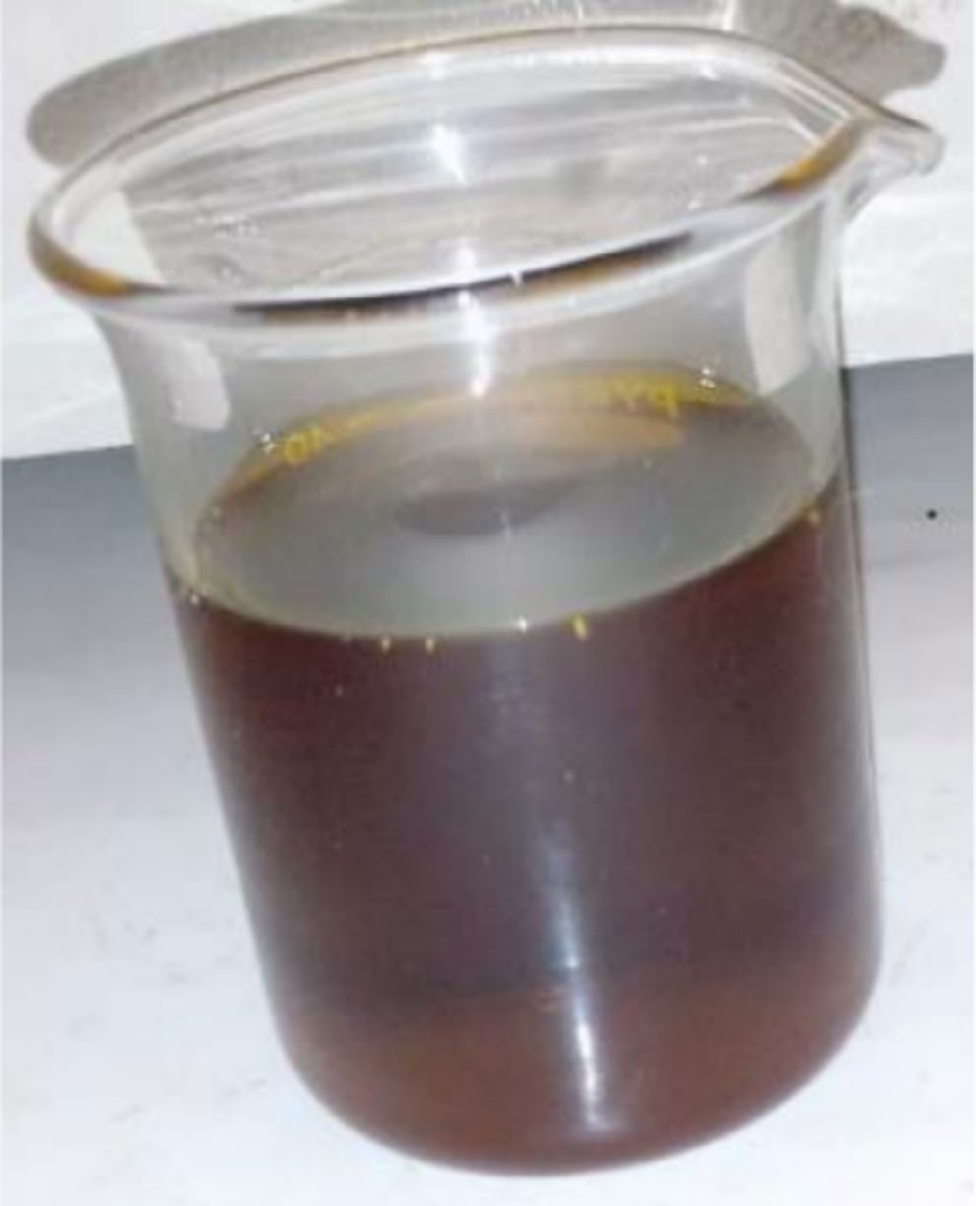
*Canarium schweinfurthii* pulp oil.

The lipid content of *C. schweinfurthii* pulp was 27.89% ± 0.26%. The oil was semi‐solid at room temperature, with a brown color and an odor characteristic of *C. schweinfurthii* fruit (Figure 2).

#### 4.1.1 Fatty Acid Profiling *C. schweinfurthii* Pulp Oil

The fatty acid profile of the oil from *C. schweinfurthii* pulp was determined and the results are given in Table 4. In general, the table showed that 23 fatty acids were present in various proportions, with a preponderance of palmitic (39.68%), oleic (28.13%), and linoleic acid (28.19%).

**TABLE 4:** Fatty acid profile of oil extracted from *C. schweinfurthii* pulp.

Fatty acids	Common name	Content (%)	Fatty acids	Common name	Content (%)
C8	Caprylic acid	0.06	C18:2n6 trans	Linoleic acid	28.19
C11	Undecanoic acid	0.01	C18:2n6 cis	Linoleic acid	0.04
C12	Dodecanoic acid	0.01	C18:3n3	α‐linolenic acid	1.43
C14	Myristic acid	0.11	C20:0	Arachidic Acid	0.06
C14:1	Myristoleic acid	0.03	C20:1n9	Eicosenoic Acid	0.07
C15:0	Pentadecanoic	0.01	C20:2	Homolinoleic acid	0.14
C16:0	Palmitic acid	39.68	C22:0	Behenic Acid	0.02
C16:1	Palmitoleic acid	0.72	C22:1n9	Éručic acid	0.01
C17:0	Margaric acid	0.07	C23:0	Tricosanoic acid	0.02
C17:1	Heptadecenoic acid	0.03	C24:0	Lignoceric acid	0.02
C18:0	Stearic acid	1.13	C22:6n3 and C24:1n9	ADH and Nervonic Acid	0.01
C18:1n9 cis and trans	Oleic acid	28.13			

The nutritional quality indices are summarized in Table 5. They provide information on the impact of *C. schweinfurthii* pulp oil on cardiovascular health and predict a real benefit to human health. The parameters PUFA/SFA, linoleic acid/α linolenic acid, atherogenic index, thrombogenic index, hypocholesterolemic/hypercholesterolemic, health‐promoting index, and nutritive value index showed respective values of 0.72, 19.74, 0.68, 0.35, 1.45, 1.46, and 40.63.

**TABLE 5:** Nutritional quality indices.

Nutritional quality indices	Content (%)	Standards
∑SFA	41.2	/
∑UFA	58.8	/
∑MUFA	28.99	/
∑PUFA	29.81	/
PUFA/SFA	0.72	> 0.45
∑(*n* − 6)	28.37	/
∑(*n* − 3)	1.44	/
Linoleic acid/α linolenic acid	19.74	> 1
Atherogenic Index (AI)	0.68	< 1
Thrombogenic Index (TI)	0.35	< 1
Hypocholesterolemic/hypercholesterolemic ratio	1.45	> 1
Health‐promoting index	1.46	> 1
Nutritive value index	40.63	> 1

#### 4.1.2 Quality Index of *C. schweinfurthii* Pulp Oil

Table 6 presents the results of the quality index for the oil extracted from the pulp of *C. schweinfurthii* . This oil showed values of 54.06 ± 0.70 g I2/100g oil; 25.80 ± 3.17 mg KOH/g; 4.02 ± 0.21 meq O_2_/kg; 0.448 ± 0.02 meq MDA/kg; 1.33 ± 0.00 and 9.37 ± 0.43, respectively for iodine, acid, peroxide, thiobarbituric acid, anisidine, and total oxidation values.

**TABLE 6:** Quality index of *C. schweinfurthii* .

Quality index	Values
Iodine value	54.06 ± 0.70 g I2/100g oil
Acid value	25.80 ± 3.17 mg KOH/g
Peroxide value	4.02 ± 0.21 meq O2/kg
Thiobarbituric acid value	0.448 ± 0.02 meq MDA/kg
Anisidine value	1.33 ± 0.00
Total oxidation value	9.37 ± 0.43

#### 4.1.3 Antiradical Activity of *C. schweinfurthii* Pulp Oil

**FIGURE 3:**
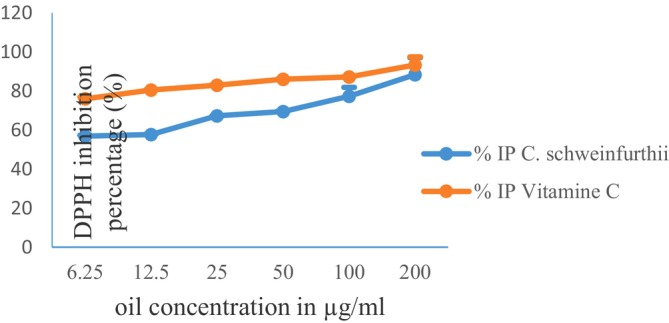
Inhibition percentage of DPPH by Vitamin C and *C. schweinfurthii* pulp oil.

Figure 3 shows the evolution of the antiradical capacity of *C. schweinfurthii* pulp oil compared with that of vitamin C. The figure shows that the percentage inhibition of DPPH increases proportionally with the concentration of oil and vitamin C (positive control). At all concentrations, vitamin C showed a higher percentage inhibition than oil.

Table 7 shows the (EC_50_) values determined from the free radical scavenging activity. It can be seen that vitamin C had a significantly lower EC_50_ (*p* < 0.05) than oil, with a value of 4.95 ± 0.18 μg/mL compared with 11.81 ± 1.99 μg/mL for oil. In addition, vitamin C exhibited significantly greater anti‐free radical activity (*p* < 0.05) than *C. schweinfurthii* oil, with values of 0.201 ± 0.15 and 0.089 ± 0.11, respectively.

**TABLE 7:** Effective concentration 50 (EC_50_) and antiradical activity (AAR) of *C. schweinfurthii* pulp oil and vitamin C.

Samples	EC50 (μg/mL)	AAR (mL/μg)
*Canarium schweinfurthii* oil	11.81 ± 1.99^b^	0.089 ± 0.15^a^
Vitamin C	4.95 ± 0.18^a^	0.201 ± 0.11^b^

*Note:* The values in the table are presented as the means ± standard deviation of 3 repetitions. For the same parameter (EC 50, AAR) and per group, values bearing different letters. a, b are significantly different (*p* < 0.05).

### 4.2 In Vivo Antihyperlipidemic Potential of *C. schweinfurthii* Pulp Oil

#### 4.2.1 Effects of *C. schweinfurthii* Pulp Oil on Lipid Profile Parameters and Atherosclerosis Index in Experimental Rats

The effects of the oil on the lipid profile of the experimental rats, in particular triacylglycerols (TAG), TC, HDL, LDL, VLDL, total lipids (TL) in addition to the atherosclerosis index (AI), were evaluated, and the results displayed in Table 8. They revealed that the consumption of the oil by the GC1 and GC2 groups of rats (male and female) decreased the TAG, TC, LDL, VLDL, TL, and AI parameters compared with the GG group, with a significant difference (*p* < 0.05). However, an increase in HDL was observed in groups GC1 and GC2 compared with the GG group, but this remained lower than that of GO2 in all experimental rats, with a significant difference (*p* < 0.05).

**TABLE 8:** Lipid profile (mg/dL) and atherosclerosis index (AI) of experimental rats.

Parameters	Sex	GS	GG	GC1	GC2	GO1	GO2
Groups							
TAG	M	113.16 ± 10.15^bB^	116.2 ± 5.40^bB^	97.63 ± 23.30^aB^	93.83 ± 1.69^aB^	97.76 ± 12.23^aB^	90.00 ± 13.20^aB^
	F	88.06 ± 5.20^cA^	100.43 ± 2.80^aA^	83.2 ± 1.68^bcA^	75.44 ± 4.57^aA^	80.49 ± 2.59^bcA^	71.13 ± 7.02^aA^
TC	M	83.77 ± 0.80^bA^	89.51 ± 3.55^cA^	79.97 ± 3.02^bA^	72.89 ± 3.59^aA^	70.93 ± 2.08^aA^	64.57 ± 0.70^aA^
	F	77.28 ± 8.77^bA^	81.55 ± 3.77^aC^	70.08 ± 8.83^bA^	62.69 ± 6.24^aB^	58.24 ± 2.72^aB^	63.52 ± 3.22^aA^
LDL	M	30.67 ± 2.14^cA^	41.75 ± 4.51^dA^	23.65 ± 2.20^cA^	15.52 ± 5.72^bA^	14.97 ± 1.88^bA^	3.83 ± 2.42^aA^
	F	28.69 ± 7.77^bA^	40.97 ± 2.88^aC^	28.04 ± 1.50^bA^	20.27 ± 4.92^bA^	10.70 ± 7.64^aA^	8.90 ± 2.13^aA^
VLDL	M	22.63 ± 1.04^aA^	23.24 ± 0.56^aA^	19.52 ± 0.33^aA^	18.76 ± 0.91^aA^	19.55 ± 0.51^aA^	18 ± 1.40^aA^
	F	17.61 ± 4.64^aA^	22.08 ± 2.03^aA^	16.64 ± 4.66^aA^	15.08 ± 1.08^aA^	16.09 ± 2.44^aA^	14.22 ± 0.33^aA^
TL	M	312.90 ± 6.55^dB^	330.23 ± 8.72^eA^	289.66 ± 9.04^cB^	268.24 ± 7.70^bB^	266.64 ± 4.83^bB^	243.61 ± 6.40^aB^
	F	274.44 ± 22.12^cA^	304.85 ± 18.37^dB^	251.78 ± 41.69^bA^	226.13 ± 18.95^aA^	219.12 ± 5.72^aA^	224.50 ± 8.60^aA^
HDL	M	30.47 ± 2.15^aA^	24.51 ± 0.87^bA^	36.79 ± 1.86^cB^	38.61 ± 1.63^cB^	36.41 ± 0.84^cA^	42.74 ± 2.71^cA^
	F	30.97 ± 4.35^cA^	18.5 ± 1.28^aA^	25.4 ± 3.81^bcA^	27.33 ± 3.28^bcA^	31.43 ± 6.86^cA^	40.39 ± 5.12^cA^
AI	M	2.74 ± 0.18^dB^	3.65 ± 0.24^eB^	2.17 ± 0.08^cA^	1.88 ± 0.17^bA^	1.94 ± 0.05^bB^	1.51 ± 0.11^aA^
	F	2.49 ± 1.16^cA^	4.40 ± 0.35^dA^	2.75 ± 0.12^cdB^	2.29 ± 0.27^abB^	1.85 ± 0.42^bA^	1.57 ± 0.15^aA^

*Note:* The values in the table are presented as the means ± standard deviation of 3 repetitions. For the same parameter (TAG, CT, HDL, LDL, VLDL, LT, or IA) and per group, values bearing different letters are significantly different (*p* < 0.05). a, b, c, d, e, ab, bc, cd = statistical analysis in all the experimental group. A, B = statistical analysis between male and female in the same experimental group.

Abbreviations: GC1 = Test animals receiving 1 mL/kg body weight of *C. schweinfurthii* oil, GC2 = Test animals receiving 2 mL/kg body weight of *C. schweinfurthii* oil, GG = Negative controls, GO1 = Positive controls receiving 1 mL/kg body weight of olive oil, GO2 = Positive controls receiving 2 mL/kg body weight of olive oil, GS = Neutral controls.

#### 4.2.2 Effect of *C. schweinfurthii* Pulp Oil on the Antioxidant Profile of Rats

Table 9 shows the effect of consumption of *C. schweinfurthii* pulp oil on the SOD and catalase activity in serum of experimental rats. The activity of the SOD and CAT enzymes in males and females fed with the hypercaloric diet GG was significantly (*p* < 0.05) reduced (55.00 ± 31.22; 41.33 ± 18.71 and 4.71 ± 2.91; 6.21 ± 0.44) compared with the GS group (76.66 ± 23.09; 82.00 ± 6.08 and 10.07 ± 0.39; 11.44 ± 0.87). However, GC2 (SOD: 85.00 ± 17.32; 91.66 ± 2.88 and CAT: 10.67 ± 0.36; 10.85 ± 0.47) showed a significant (*p* < 0.05) increase in the activity of these enzymes compared with the GG group.

**TABLE 9:** Antioxidant power of *C. schweinfurthii* pulp oil in experimental male and female rats.

Parameters	Sex	GS	GG	GC_1_	GC_2_	GO_1_	GO_2_
Groups							
SOD (%)	M	76.66 ± 23.09^aA^	55.00 ± 31.22^bB^	76.66 ± 12.58^aA^	85.00 ± 17.32^aA^	81.66 ± 20.20^aA^	91.66 ± 5.77^aA^
	F	82.00 ± 6.08^aA^	41.33 ± 18.71^bC^	86.66 ± 7.63^aA^	91.66 ± 2.88^aA^	91.66 ± 5.77^aA^	93.33 ± 7.63^aA^
CAT (μM/min/mL)	M	10.07 ± 0.39^aA^	4.71 ± 2.91^bB^	10.48 ± 1.35^aA^	10.67 ± 0.36^aA^	10.63 ± 1.46^aA^	10.97 ± 0.45^aA^
	F	11.44 ± 0.87^aA^	6.21 ± 0.44^bB^	10.65 ± 0.05^aA^	10.85 ± 0.47^aA^	10.69 ± 1.32^aA^	12.37 ± 0.37^aA^

*Note:* The values in the table are presented as the means ± standard deviation of 3 repetitions. For the same parameter (SOD or CAT) and per group, values bearing different letters are significantly different (*p* < 0.05). a, b = statistical analysis in all the experimental group. A, B = statistical analysis between male and female in the same experimental group.

Abbreviations: GC1 = Test rats receiving 1 mL/kg body weight of *C. schweinfurthii* oil, GC2 = Test rats receiving 2 mL/kg body weight of *C. schweinfurthii* oil, GG = Negative controls, GO1 = Positive controls receiving 1 mL/kg body weight of olive oil, GO2 = Positive controls receiving 2 mL/kg body weight of olive oil, GS = Neutral controls.

## 5 Discussion

### 5.1 Lipid Profile of *C. schweinfurthii* Pulp Oil

In this study, the lipid content of *C. schweinfurthii* fruit pulp was 27.89%. This value was lower than the 38% reported by Kapseu (2009) for the same part of the fruit from northern Cameroon. This difference could be attributed to the agroclimatic and agrosoil factors to which the plant was subjected, as well as its stage of ripening. Indeed, Gigon and Le Jeune (2010) and Nkouam (2018) state that the different constituents of a plant and their content depend on the stage of maturity, the variety, the growing area, and local agronomic practices.

The fatty acid composition of oil influences its therapeutic potential in the prevention and treatment of cardiovascular diseases through beneficial or harmful effects. The PUFA of this oil is strongly reflected by bioactive lipids which have health benefits such as anti‐inflammatory, neuroprotective, hypoglycemic, hypotensive, and hypolipidemic actions (Mbundu et al. 2018; Nameni et al. 2021). SFA could increase the risk and progression of metabolic diseases by increasing serum cholesterol levels, while PUFA could be beneficial to health by increasing the activity of low‐density lipoprotein receptors and reducing serum cholesterol concentration (Chen and Liu 2020).

The fatty acid profile of *C. schweinfurthii* pulp oil consisted of 23 fatty acids with a preponderance of palmitic (39.68%), oleic (28.13%), and linoleic (28.19%) acids. The proportions obtained by Anyalogbu Ernest et al. (2017) whose matrix was collected in Nigeria were different.

Several nutritional indices linked to fatty acid profiles have been proposed to assess the impact of diet on cardiovascular health, in particular the effect of certain fatty acids on cholesterol metabolism and dyslipidemia. A PUFA/SFA ratio greater than 0.45 is recommended in the human diet to prevent diseases (Rahman et al. 2023). In this study, this ratio was 0.72, which could suggest that this oil is capable of reducing the risk of cardiovascular disease and other chronic illnesses.

The Linoleic acid/α linolenic acid ratio was developed for guiding infant formula (Chen and Liu 2020). A high value greater than 1 such as in this study (19.74) is highly appreciated in baby food and infant formula (Chen and Liu 2020). Atherogenic Index (AI) less than 1 is good for human health (Rahman et al. 2023). The value obtained in this study was 0.68, which is recommended for positive health benefits. Thrombogenic index indicates the capacity of oil to prevent plaque accumulation and coronary diseases. The value < 1 like in this study (0.35) is recommended and suggests high nutritional value of fatty acids and permits the reduction of risks of dyslipidemia (Mgbechidinma et al. 2023). Hypocholesterolemic/hypercholesterolemic ratio evaluates the influence of specific fatty acids on cholesterol metabolism and cardiovascular diseases (Chen and Liu 2020). The value 1.45 obtained in this study is desirable for higher nutritional value because, standards recommend a value greater than 1 for positive health benefits (Mgbechidinma et al. 2023). Health‐promoting index was proposed to evaluate the effect of fatty acid composition on cardiovascular risks. The value obtained in this study was 1.46. It has been specified that a higher value of this parameter is more beneficial to human health (Chen and Liu 2020; Rahman et al. 2023). The nutritive value index is another indicator of the effect of fatty acids on cholesterol metabolism. A higher value such as that obtained in this study (40.63) is more beneficial for human health because, it is linked to a low proportion of SFA and a high proportion of UFA which, on the other hand, improves blood cholesterol regulation and is involved in the prevention of CVD (Mgbechidinma et al. 2023).

### 5.2 Quality Indices of *C. schweinfurthii* Pulp Oil

The iodine value provides information on the degree of unsaturation of the oil. A high iodine value indicates that the oil is rich in fatty acids that are easily oxidized. In such circumstances, the oil should not be heated to high temperatures. However, a low value could indicate oxidation or a high content of SFAs that are resistant to oxidation (Ntube et al. 2024). The iodine value of *C. schweinfurthii* pulp oil was 54.06 ± 0.70 g I2/100g oil. This value is higher than that of Agbo et al. (1992) who obtained a value of 36.00 ± 3.00 g I2/100g oil. This variation could be due firstly to the presence in this oil of oleic, linoleic and α‐linolenic acids whose unsaturation has been preserved. In fact, the fruit used in the study of Agbo et al. (1992) came from Côte d'Ivoire and the oil was obtained by a process that required pulp drying at 70°C in addition to extraction by the Soxhlet method followed by evapoconcentration at 70°C. However, the pulps in this study were dried at 45°C, and the oil was extracted using the hexane maceration method at room temperature, followed by evapoconcentration at 50°C, which could have preserved the UFA from oxidation (Saleh‐E‐In and Royb 2007). Nevertheless, the iodine value obtained in this study was within the standard proposed by the Codex Alimentarius (1999), which stipulates that a good quality oil should have an iodine value of less than 56 g I2/100g oil.

The acid value provides information on the level of free fatty acids present in an oil and is a relative measure of its rancidity (Lopez et al. 1997). In this study, the value of 25.80 ± 3.17 mg.

KOH/g was higher than 10.2 ± 0.2 mg KOH/g obtained by Kapseu (2009). This free acidity could be explained by the oxidation of TAGs releasing free fatty acids into the oil AFNOR (Association Française de Normalisation) (1981). However, this value is lower than that of Ajiwe et al. (1998) which was 32.48 ± 0.8 mg KOH/g. In fact, a short time between harvesting, extraction and storage in the dark could be factors that limited the photooxidation and lipolytic action of the enzymes observed during the storage of plant matrices (Nkouam 2018). However, the value obtained deviates from the proposed Codex Alimentarius (2009), which specifies that a good quality oil should have an acid number of less than 4 mg KOH/g.

The peroxide value indicates the degree of primary oxidation of an oil. The value obtained was 4.02 ± 0.21 meq O2/kg. This value is higher than that obtained by Aboubakar Dandjouma et al. (2008), whose value was 3.40 ± 0.22 meq O2/kg. This variation could be attributed to oxidation of the oil by the formation of primary oxidation compounds, which are none other than hydroperoxides (Eymard 2003). Also, these results do not corroborate those reported by Kapseu (2009), whose value obtained was 7.8 ± 0.1. This difference could be due to the decomposition of hydroperoxides in favor of secondary oxidation compounds such as 2‐alkenals and 2,4‐dienals (Ampem et al. 2024). However, the peroxide value obtained remains within the standard proposed by Codex Alimentarius (1999), which recommends that a good quality oil should have a peroxide value of less than 10 meq O2/kg.

The thiobarbituric acid value provides information on the presence of secondary oxidation products responsible for the rancidity of lipids, particularly the MDA produced during the oxidation of PUFAs (Esfarjani et al. 2019). Its value in this study was 0.44 ± 0.02 meq MDA/kg, corresponding to the standard recommended by the Codex Alimentarius (1999). This standard specifies a value of less than 2 meq MDA/kg for this parameter. This result can be explained by the presence in *C. schweinfurthii* oil of compounds with antioxidant activity, such as phenolic compounds, which would be entrained during oil extraction (Liu et al. 2022). The latter would have limited the formation of hydroperoxides and consequently their conversion into oxidation by‐products by giving up their hydrogen atom, thus enabling them to preserve their double bonds and therefore the quality of the oil (Dongmo et al. 2010).

The anisidine value measures the aldehyde content, mainly 2‐alkenals and 2,4‐dienals, which are secondary oxidation compounds generated during the decomposition of hydroperoxides in oils, often characteristic of their rancidity (Esfarjani et al. 2019). The value obtained in this work was 1.33 ± 0.00. This value is justified by the low transformation of hydroperoxides into secondary oxidation compounds, the fact that *C. schweinfurthii* oil is not subjected to high temperatures, and the antioxidant activity of the phenolic compounds present, which would have slowed the decomposition of the hydroperoxides formed (Roman 2012). Furthermore, according to the Codex Alimentarius (2015), this oil is within the standard because its anisidine index is < 20.

The total oxidation value measures both primary and secondary oxidation products, specifically hydroperoxides and their decomposition products (Shahidi and Wanasundara 2008). Determination of this parameter yielded a value of 9.37 ± 0.43; this shows that the oil is oxidized despite the fact that it remains within the standard proposed by the Codex Alimentarius (2015), which recommends that it be less than or equal to 26.

### 5.3 Antioxidant Activity of *C. schweinfurthii* Pulp Oil

DPPH. is a free radical widely used as a molecule for estimating the free radical scavenging activity of antioxidant compounds. It accepts an electron or proton to become a stable molecule. The ability to trap the DPPH. radical is determined by the decrease in absorbance at 517 nm induced by the antioxidant (Sanchez‐Moreno 2002). The antioxidant capacity of *C. schweinfurthii* oil was lower than that of vitamin C at all concentrations. This could be explained by its structure, which favors the departure of several protons to stabilize the free radical. This makes it a benchmark antioxidant, unlike other antioxidants which have few labile protons. The EC_50_ value determined from the antiradical activity gave 11.81 ± 1.99 μg/mL for *C. schweinfurthii* which is higher than 4.95 ± 0.18 μg/mL for vitamin C. Indeed, according to the classification of Souri et al. (2008), for an EC_50_ < 20 μg/mL, the antioxidant activity is strong; for 20 μg/mL < EC50 < 75 μg/mL, the antioxidant activity is moderate; and for EC_50_ > 75 μg/mL the antioxidant activity is weak. This means that *C. schweinfurthii* oil has strong antioxidant activity, but less than that of vitamin C. This could be explained by the structure of phenolic compounds, which favored the departure of several protons to stabilize the free radical. This makes it a benchmark antioxidant, unlike other antioxidants which have few labile protons. Nevertheless, this activity of *C. schweinfurthii* oil could be explained by the presence of phenolic compounds that were carried away by the solvent during lipid extraction (Hein et al. 2012). This result corroborates that of Wahab et al. (2015), who showed that antioxidant activity is not only dose‐dependent but also structure‐dependent, favoring the departure of protons to stabilize free radicals.

### 5.4 In Vivo Antihyperlipidemic Potential of *C. schweinfurthii* Pulp Oil

#### 5.4.1 Effects of *C. schweinfurthii* Pulp Oil on Lipid Profile Parameters and Atherosclerosis Index in Experimental Rats

The lipid profile refers to the concentration of circulating lipid fractions. It is assessed in the fasting state and provides information on lipid status by measuring TAG, TC, HDL, LDL, VLDL, and LT. Imbalance between these parameters predisposes to CVD, one indicator of which is the atherosclerosis index (AI) (Atsafack et al. 2015).

The results obtained in this study showed that the TAG concentration is higher in male and female rats in the GG group (116.2 ± 5.40 and 100.43 ± 2.80) compared with those fed only the basic GS diet (113.16 ± 10.15 and 88.06 ± 5.20). This high level is thought to be due to the hypercaloric diet rich in SFAs administered to the GG group, which allowed them to accumulate. This result corroborates those of Takam et al. (2019) who showed that the hyperlipidemic diet increases serum TAG levels. However, these data also indicate a significant decrease in serum TAG concentration in all male and female rats in the GC2 group compared with the GG group. This indicates that regular consumption of *C. schweinfurthii* pulp oil leads to a reduction in serum TAG concentration. In addition, as this oil is a source of omega 3 and 6, it is thought to have a TAG‐reducing effect. The presence of linoleic and α‐linolenic acids is thought to have a powerful inhibitory effect on hepatic TAG synthesis via inhibition of enzymes such as diacylglycerol acyltransferase and acetylCoA carboxylase. This inhibition was accompanied by an increase of ß‐oxidation in hepatic mitochondria and peroxisomes, leading to a reduction in substrate availability for TAG synthesis. This fall in TAG levels could also refer to a reduction in postprandial lipid influx due to α‐linolenic acid, suggesting a reduction or delay in fat absorption (Fokou et al. 2009; Mozaffarian and Wu 2011). These results are also in line with those of Nameni et al. (2021) who showed that *Citrullus lanatus* and *Cucumeropsis mannii* oil significantly reduced TAG levels in dyslipidemic rats.

The significant difference (*p* < 0.05) observed between males and females in all groups can be attributed to the hormonal variations generally observed in females which synthesize estrogens from fatty acids thus modifying the lipid profile towards an antiatheromatous profile. In particular, they reduce TC and TAG levels and increase the resistance of LDL to oxidation (Manassier 2013).

The results show that TC concentration is significantly higher (*p* < 0.05) in male and female rats in the GG disease group (89.51 ± 3.55 and 81.55 ± 3.77) compared with the GS neutral control (83.77 ± 0.80 and 77.28 ± 8.77). This is thought to be due to increased uptake of exogenous cholesterol, subsequent deposition and decreased cholesterol catabolism leading to reduced bile acid production and turnover (Kalaivani et al. 2017). However, the results of this study also show a significant reduction (*p* < 0.05) in serum TC levels in rats treated with *C. schweinfurthii* oil at a dose of 2 mL/kg body weight (72.89 ± 3.59 and 62.69 ± 6.24). This reduction is thought to be in response to the presence of linoleic acid and phytosterols known for their cholesterol‐lowering activity by reduction of cholesterol biosynthesis through inhibition of 3‐hydroxy‐3‐methylglutaryl coenzyme A (HMGCoA) reductase, promoting fecal excretion of bile acids and neutral sterols (Khairi et al. 2014; Okediran et al. 2015). These results are similar to those of Eke et al. (2021) and Nguekouo et al. (2018) who showed respectively that *Citrullus lanatus seeds* oil and *Abelmoschus esculentus* fruit lowered TC levels.

A significant decrease (*p* < 0.05) in LDL levels following administration of *C. schweinfurthii* pulp oil to the GC2 group (15.52 ± 5.72 and 20.27 ± 4.92) compared with rats fed the hypercaloric GG diet (41.75 ± 4.51 and 40.97 ± 2.88) was observed in this study. This effect was also observed after consumption of *Citrullus lanatus seeds* oil (Eke et al. 2021). This result can be explained by the similarity in the composition of *C. schweinfurthii* oil and the olive oil used as a control, particularly with the presence of the PUFAs Ѡ3 and Ѡ6. These fatty acids reduce LDL‐C by inhibiting the HMG‐CoA reductase enzyme, leading to a reduction in hepatic cholesterol synthesis. It increases the synthesis of LDL‐C receptors in hepatocytes and increases their uptake from the circulation to replace intracellular cholesterol (Okediran et al. 2015). In addition, these PUFAs also have an antioxidant effect that modulates the expression or activity of molecules involved in the atherosclerotic process, thereby reducing CVD risk factors such as hyperlipidaemia (Kermanshahi Pour et al. 2017). These results are in line with those of Kengne et al. (2020), who obtained similar effects with *Raphia hookeri* oil.

A significant increase (*p* < 0.05) in total lipids was observed in rats fed the hypercaloric diet (330.23 ± 8.72 and 304.85 ± 18.37) compared with the healthy group (312.90 ± 6.55 and 274.44 ± 22.12). This would be due to an exogenous intake of lipids accompanied by a significant increase in LDL at the high reduction in HDL levels, pointing to a model of dietary hyperlipidemia (Cohen et al. 2005).

A high concentration of HDL is antiarteriogenic, reason why its reduction is associated with an increased risk of CVD (Prabhavathi Devi et al. 2018). A significantly (*p* < 0.05) high level of HDL in rats treated with *C. schweinfurthii* pulp oil at a dose of 2 mL/kg body weight (38.61 ± 1.63 and 27.33 ± 3.28) was observed in this study. This could be explained by the presence of oleic and linoleic acids in this oil, which stimulated HDL production by the degradation of serum TC. In addition, the lower SFA level permits a good HDL metabolism which signifies a higher level of these lipoproteins (Katare et al. 2011). These results corroborate those of Al‐Okbi et al. (2014) who showed that *Cucurbita pepo* oil increased HDL levels in dyslipidemic rats.

The TC/HDL ratio represents the atherosclerosis index (AI). In this study, the index was significantly higher (*p* < 0.05) in rats fed the high‐calorie diet (3.65 ± 0.24 and 4.40 ± 0.35) than in rats fed with standard diet (2.74 ± 0.18 and 2.49 ± 1.16). In clinical interpretation of these parameters, the TC/HDL ratio should be between 2.5 and 3. This ratio (AI) is greater than 3 in rats fed with the hypercaloric diet could confirm the establishment and evolution of the dyslipidemic and arteriosclerotic process (Njinkoue et al. 2017). The administration of *C. schweinfurthii* oil at a dose of 2 mL/kg body weight significantly (*p* < 0.05) reduced AI (1.88 ± 0.17 and 2.29 ± 0.27). This suggests that *C. schweinfurthii* oil helps to regulate lipid profile parameters in the direction of their return to normal ranges due to oleic and linolenic acids (Achu et al. 2016).

#### 5.4.2 Effect of *C. schweinfurthii* Pulp Oil on the Antioxidant Profile of Rats

Dyslipidemia leads to an increase in the production of free radicals, which also play an important role in the onset of CVD (Jiangwei et al. 2011). The effects of *C. schweinfurthii* oil were assessed on the antioxidant profile of experimental rats.

The results obtained from this study showed a significant decrease (*p* < 0.05) in SOD enzyme activity in male and female rats fed the high‐calorie diet compared with those fed the standard diet (76.66 ± 23.09; 55.00 ± 31.22 and 82.00 ± 6.08; 41.33 ± 18.71) and catalase (10.07 ± 0.39; 4.71 ± 2.91 and 11.44 ± 0.87; 6.21 ± 0.44). This could be due to the hypercaloric diet administered to the GG rats (Mir et al. 2014). In contrast, following consumption of *C. schweinfurthii* oil, an increase in the activity of each of these enzymes was observed. This result could be explained by the frequent consumption of *C. schweinfurthii* . This oil is thought to contain antioxidant compounds that would have neutralized reactive oxygen species. These antioxidants would have activated the nuclear transcription factor of antioxidant enzymes and consequently their overexpression, with an improvement in the antioxidant balance and therefore an increase in their activity (Eggler et al. 2008). These results are similar to those of Athmani (2016) and Mir et al. (2014) who, after subjecting hypercholesterolemic rats to a diet based on proteins or their hydrolysates, showed an increase in the activity of antioxidant enzymes in various tissues.

## 6 Conclusion

This study aimed to assess the antihyperlipidemic potential of *Canarium schweinfurthii* Engl. of pulp oil. The studies showed that this oil is of good quality according to pre‐established standards for vegetable oils. It contains a high proportion of palmitic, oleic, and linoleic acids and has strong in vitro antioxidant capacity. Daily intake of *C. schweinfurthii* pulp oil modulates lipid metabolism by significantly reducing serum TAG, TC, LDL, and VLDL levels and increasing serum HDL concentration in rats. It has an antiatherogenic effect and increases the activity of the serum antioxidant enzymes SOD and catalase in rats. *Canarium schweinfurthii* fruit pulp oil can be used to prevent and fight against dyslipidemia in rats.

## Author Contributions

**Archelle Arnellie Abaoabo Foudjin:** investigation (equal), writing – original draft (equal). **Hermine Tsafack Doungue:** validation (equal), writing – review and editing (equal). **Stephano Tambo Tene:** formal analysis (equal). **Ronice Zokou:** data curation (equal), software (equal). **Geradin Joel Tagne Tueguem:** methodology (equal). **Tekou Florian Amel:** methodology (equal). **Anne Pascale Kengne Nouemsi:** conceptualization (equal), project administration (equal), supervision (equal).

## Funding

The authors have nothing to report.

## Ethics Statement

Animals were treated in accordance with OECD principles, which prioritize animal welfare throughout the study. The number of rats used in the experiments was limited to the minimum necessary, and methods were employed to minimize suffering and pain. An ethical clearance for animal handling and care was obtained from the university of Buea‐institutional animal care and use committee permit number: UB‐IACUC NO 01/2024.

## Conflicts of Interest

The authors declare no conflicts of interest.

## Data Availability

The data that support the findings of this study are available on request from the corresponding author. The data are not publicly available due to privacy or ethical restrictions.
